# Supplementary material for: Improved Differentiation Towards Insulin Producing Beta-Cells Derived from Healthy Canine Pancreatic Ductal Organoids
Source: Vet Sci. 2025 Apr 13;12(4):362. doi: 10.3390/vetsci12040362 (PMC12030824; doi:10.3390/vetsci12040362)
Supplement: Supplementary file 1 [file vetsci-12-00362-s001.zip › vetsci-3484755-supplementary.pdf]

Supporting information

Supplementary Tables

Donor ID	Gender	Date of Birth	Date of death	Weight (kg)	Breed	Application
176002	Female	21-05-2018	27-01-2020	18	Beagle	Cell culture
178820	Female	18-06-2018	27-01-2020	19	Beagle	Cell culture
531104	Female	18-12-2016	20-11-2020	10	Bastard	Cell culture
168646	Female	06-02-2018	28-05-2019	22	Beagle	Cell culture
215768	Male	03-12-2019	18-05-2021	27	Bastard	Islet qPCR

Supplementary Table S1. *Donor information used for cell culture, islet isolation or tissue qPCR.*

dpEM		PDM-A		PDM-B	
**Compound**	**Final concentration**	**Compound**	**Final concentration**	**Compound**	**Final concentration**
A/D +	-	A/D +	-	A/D +	-
WCM (wnt3a)	30%	BSA	2 g/l	BSA	2 g/l
R-spondin3	10%	N2	1%	ITS	25 µg/mL
B27 (w/o vit A)	1%	ITS	25 µg/mL	NAC	1.25 mM
N2	1%	NAC	1.25 mM	SANT-1	0.25 µM
NIC	10 mM	RA	100 nM	RA	50 nM
NAC	1.25 mM	NIC	1 mM	A83-01	10 µM
FGF-10	100 ng/mL	SANT-1	0.25 µM	T3	1 µM
Gastrin	10 nM	Noggin	50 ng/mL	NIC	1 mM
Noggin	100 ng/mL	Primocin	50 µg/mL	Noggin	50 ng/mL
EGF	50 ng/mL	TPPB	0.2 µM	Primocin	50 µg/mL
Primocin	50 µg/mL	FGF-10	50 ng/mL	Rock inhibitor	10 µM
A83-01	0.5 µM	EGF	50 ng/mL	Forskolin	10 µM
				Exendin-4	10 nM
				TPPB	0.2 µM

PDM-C		PDM-D1		PDM-D2	
**Compound**	**Final concentration**	**Compound**	**Final concentration**	**Compound**	**Final concentration**
A/D +	-	ELGM1	-	ELGM2	-
BSA	2 g/l	BSA	20 g/l	BSA	20 g/l
NIC	10 mM	Betacellulin	10 ng/mL		
Glucose additive	1.35 g/l	NIC	10 mM		
ITS	25 µg/mL	NAC	1.25 mM		
NAC	1.25 mM	Exendin-4	10 nM		
Retinoic acid	0.1 µM	T3	1 µM		
A83-01	10 µM	ITS	25 µg/mL		
T3	1 µM	Activin A	100 ng/mL		
Noggin	50 ng/mL	Matrigel	0.18mg/mL		
Primocin	50 ug/mL				
Rock inhibitor	10 µM				
Forskolin	10 µM				
Exendin-4	10nM				
SANT-1	0.25 µM				
Latrunculin A	1 µM				

ALK5iII	10 µM
DAPT	1 µM
Wnt5a	100 ng/mL
PF573228	3 µM

Supplementary Table S2. *Combined overview of expansion, enhanced differentiation and maturation media.*

Product gene of PCR	Abbreviation	Marker for	Genebank genomic ID	Genebank accession number mRNA	Forward Primer	Reverse Primer	Temperature (°C)	Product size (bp)
Pancreatic and duodenal homeobox 1	PDX1	Multipotent / pancreatic precursor, mature β cell	493994	NM_001284471.2	GATGAAGTCTACCAAGGCTCAC	GTTGAACAGGAACTCCTTCTCCA	63-65	140
NK6 Homeobox 1	NKX6.1	Pancreatic precursor, mature β cell	487838	XM_038443803.1	GCAGATCTTCGCTCTGGA	GAACCAGACCTTGACCTGAC	63-65	114
Solute Carrier Family 2 Member 2 / Glucose Transporter Type 2	GLUT2	Mature β cell	488165	XM_014110374.3	GCTCTGGTCCTTGTCTGTG	GCTTTGACTCTTCCAATCCGA	51.5-55	95
Insulin	INS	Mature β cell	483665	NM_001130093.2	GCTGGAGAATTACTGCAACTAGG	CAGAGGGTTTATTGAATCACTTG	60-63	118
Proprotein convertase subtilisin/kexin type 1	PCSK1	Mature β cell	479149	XM_038660778.1	TCTGGAAGCAAATCCAAATCTC	TTAGCAACCCAAATCCAAATCG	58.5-63.5	148
Glucagon	GCG	Mature α cell	403571	NM_001003044.1	GTCATTCTCAGGGTACATTCAC	GGCAATGTTATTCTTGTTCCTC	64	113
Somatostatin	SST	Mature δ cell	403993	NM_001003307.1	TCTGCAGAAGTCCCTGGCT	CAGCCTCATTTCATCCTGCTC	61	150
SRY-Box Transcription Factor 9	SOX9	Trunk bipotent precursors, mature ductal cell	403464	NM_001002978.1	CTCAGCGTCTTCACCTCCT	TGGGAATGTATGTCAAAGCGT	68	105
Mucin 1	MUC1	Mature ductal cell	448784	NM_001194977.1	ACTGTTCCACCTCCTCCCA	GATGAGTTGCCTCCCTGTGC	67	115
Alpha-Amylase	a-AMY	Exocrine cell	607460	XM_038669971.1	TGGAAGTTACTTCAACCCTGG	CGACAATCTCTGACCTGATACG	61	137
Marker of proliferation KI-67	KI67	Proliferation	100686578	XM_038440933.1	AATCATCAAGGAATACCCTCCA	GGAGAAGTTTGCATAGGATCAC	62	120
Proliferating cell nuclear antigen	PCNA	Proliferation	477166	XM_038433208.1	GCAAGTGGAGAACTAGGAAATGG	CATCTCTATGGTAACAGCTTCCTC	64	87
Leucine Rich Repeat Containing G Protein-Coupled Receptor 5	LGR5	Stem cell	609478	XM_038678819.1	CTCAGCGTCTTCACCTCCT	TGGGAATGTATGTCAAAGCGT	61	139
Ribosomal protein S5	RPS5	Reference gene	476366	XM_038655655.1	TCACTGGTGAGAACCCCCT	CCTGATTCACACGGCGTAG	62.5	141
Signal Recognition Particle Receptor	SRPR	Reference gene	489293	XM_038664445.1	GCTTCAGGATCTGGACTGC	GTTCCCTTGGTAGCACTGG	61	81
Succinate Dehydrogenase Complex Flavoprotein Subunit A	SDHA	Reference gene	478634	XM_038445573.1	GCCTTGGATCTCTTGATGGA	TTCTTGGCTCTTATGCGATG	61	92

Supplementary Table S3. *Validated primers used for qPCR*
